# Prevalence of malocclusions in 8- and 9-year-old children in Germany—Results of the Sixth German Oral Health Study (DMS 6)

**DOI:** 10.1007/s00056-022-00437-z

**Published:** 2023-02-01

**Authors:** Andreas Rainer Jordan, Kathrin Kuhr, Nicolas Frenzel Baudisch, Christian Kirschneck

**Affiliations:** 1 Institute of German Dentists, Universitätsstr. 73, 50931 Cologne, Germany; 2 Department for Orthodontics, University Hospital Regensburg, Franz-Josef-Strauß-Allee 11, 93053 Regensburg, Germany

**Keywords:** Index of Complexity, Outcome and Need, Epidemiology, Orthodontics, KIG classification, Health care research, Index of Complexity, Outcome and Need, Epidemiologie, Kieferorthopädie, KIG-Klassifikation, Versorgungsforschung

## Abstract

**Purpose:**

Current population-wide data on the prevalence of malocclusions in 8‑ and 9‑year-old children in Germany are not available. Therefore, the primary objective of this study was to collect data on the prevalence of malocclusions in 8‑ and 9‑year-old children in Germany. The secondary objective of this study was to use this information to derive the need for orthodontic care provision.

**Methods:**

This is an oral–epidemiological investigation and social science survey at the national level with a focus on tooth and jaw misalignment. The investigation took place between January and March 2021 at 16 study centers across Germany. All relevant data were available for the 705 study participants and were included in the statistical analysis.

**Results:**

Overbite was the most common finding with 88.9%. Also widespread were crowding, with at least 60.9%, and lack of space, with a share of 30.9%. All other indication groups had a share below 10%. Rare (< 1%) were buccal and lingual occlusions and craniofacial abnormalities. The most severe forms of disease (Orthodontic Indication Group [Kieferorthopädische Indikationsgruppen, KIG] grade 5) were overbite (3.2%), open bite malocclusion (1.0%), undershot (0.6%), and craniofacial abnormalities (0.4%). The proportion of study participants who required orthodontic treatment, in accordance with statutory health insurance provider guidelines, was 40.4%. The proportion of study participants in principle requiring orthodontic treatment for medical reasons was 97.5%. Systemic differences in the need for orthodontic care provision relating to gender, region, or social status were not identified.

**Conclusion:**

In general, the need for care provision identified in the orthodontic indication groups corresponds to that shown in previous studies. This suggests that the need for orthodontic treatment in Germany has remained stable over the years.

**Supplementary Information:**

The online version of this article (10.1007/s00056-022-00437-z) contains the German version of this article as supplementary material, which is available to authorized users.

## Abstract (de)

**Ziel:**

Aktuelle, bevölkerungsweite Daten zur Verbreitung von Zahn- und Kieferfehlstellungen in Deutschland liegen nicht vor. Es war daher das primäre Ziel dieser Studie, die Verbreitung von Zahn- und Kieferfehlstellungen bei 8‑ und 9‑jährigen Kindern in Deutschland zu erfassen. Es war das sekundäre Ziel dieser Studie, daraus den kieferorthopädischen Versorgungsbedarf abzuleiten.

**Methode:**

Es handelt sich um einen oralepidemiologischen Untersuchungs- und sozialwissenschaftlichen Befragungssurvey auf national repräsentativer Ebene mit Scherpunkt auf Zahn- und Kieferfehlstellungen. Die Untersuchungen fanden von Januar bis März 2021 in 16 Studienzentren in Deutschland statt. Für 705 Studienteilnehmende lagen alle relevanten Daten vor, sie wurden in die statistische Auswertung einbezogen.

**Ergebnisse:**

Am häufigsten kamen mit 88,9 % Überbisse vor. Ebenfalls weit verbreitet waren Engstand mit mindestens 60,9 % sowie Platzmangel mit einem Anteil von 30,3 %. Alle anderen Indikationsgruppen wiesen jeweils einen Anteil von unter 10 % auf. Selten (< 1 %) wurden Bukkal‑/Lingualokklusionen sowie kraniofaziale Anomalien vorgefunden. Die schwerwiegendsten Erkrankungsformen (KIG [Kieferorthopädische Indikationsgruppen] Grad 5) stellten mit 3,2 % der Überbiss, mit 1,0 % der offene Biss, mit 0,6 % der Vorbiss und die kraniofazialen Anomalien (0,4 %) dar. Der Anteil der Studienteilnehmenden, bei denen nach den Richtlinien der gesetzlichen Krankenversicherung eine kieferorthopädische Behandlung angezeigt ist, lag bei 40,4 %. Der Anteil der Studienteilnehmenden, bei denen aus medizinischen Gründen eine kieferorthopädische Behandlung grundsätzlich angezeigt war, lag bei insgesamt 97,5 %. Systematische Unterschiede im Hinblick auf das Geschlecht, die Region oder den Sozialstatus wurden beim Versorgungsbedarf nicht festgestellt.

**Schlussfolgerungen:**

Der im Rahmen dieser Studie nach KIG ermittelte Versorgungsbedarf deckt sich weitgehend mit dem in früheren Untersuchungen dargestellten. Damit liegt nahe, dass der kieferorthopädische Behandlungsbedarf in Deutschland über die Jahre weitgehend konstant geblieben ist.

## Introduction

Alongside caries and periodontal diseases, tooth and jaw misalignment are among the most common health problems affecting the oral cavity [1]. Diseases of the masticatory system, i.e., teeth, jaw, temporomandibular joint, and masticatory muscles, can seriously affect well-being and quality of life, causing pain and suffering, affecting food intake or food choice, and making speech difficult [2]. In this sense, orthodontics is heavily orientated towards prevention when orthodontic treatment can prevent the onset of sequelae. It is known that orthodontic abnormalities are associated with impairment of masticatory function [3], breathing [3, 4], phonetics, and swallowing [5, 6], and an enlarged overjet significantly increases the risk of trauma to the front teeth [7] and orthodontic overjet correction can effectively reduce this risk [8].

The causes of orthodontic diseases are multifactorial and range from genetic, epigenetic, and functional factors to environmental factors. The severity of each individual disease is highly variable. Correspondingly, the range of therapeutic options is extensive. The influence of orthodontic treatment on genetic and epigenetic factors is limited; treatment tends to primarily focus on the consequences of these factors. However, in principle, there are preventive options for functional and environmental factors, and often also causal therapeutic options.

Traditionally, tooth and jaw misalignment were classified based on the malocclusion status of the 6‑year molars, known as Angle’s classification, and the results were used to determine the position of the jaws in relation to one another. The distribution of Angle’s classification varies greatly from region to region, although globally all Angle’s classifications are represented [1].

In permanent dentition, the prevalence of Angle class I globally is approximately 75%, followed by Angle class II at approximately 20%. Angle class III has a proportion of approximately 6%. An orthodontic–epidemiological study of 494 9‑year-olds in southwest Germany also found that Angle class I was the most prevalent in children, followed by Angle classes II and III [9]. In the same study, Angle class II dentitions were observed in approximately 20%; this value is within the variance range of the prevalences reported in 2018 by Alhammadi et al. [1]. Angle class II was observed in 3% of those examined. An epidemiological–orthodontic study conducted as part of school dental examinations in Frankfurt am Main on 1251 school pupils aged between 9 and 11 years analyzed the results in accordance with the diagnostic chart of the statutory health insurance providers in Germany; the Orthodontic Indication Groups (Kieferorthopädische Indikationsgruppen, KIG) [10].

This study found that treatment was indicated, in accordance with the statutory health insurance provider guidelines (KIG ≥ 3), in 41.4% of all examined cases. Stahl et al. discovered that habits, dysfunction, and dyskinesia affecting deciduous to mixed dentition increased significantly [11]. Oral habits were observed more frequently in girls than in boys, whereas articulation disorders were more prevalent in boys.

Overall, it was determined that myofunctional disorders are more prevalent in children with greater sagittal overjet, open bite malocclusions, lateral crossbite, and progenia (Angle class III). A further report from the same team of authors observed physiological occlusal relationships in one-quarter of children. The number dropped significantly to 7% when children with mixed dentition were examined [12].

The First German Oral Health Study (Erste Deutsche Mundgesundheitsstudie, DMS 1) conducted by the Institute of German Dentists (Institut der Deutschen Zahnärzte, IDZ) in 1989 laid the foundation for population-representative social–epidemiological monitoring of oral health and oral health care provision in Germany [13]. Previously, tooth and jaw misalignment had only been investigated in the former West German states during the First German Oral Health Study in 1989. Current population-wide data on the prevalence of tooth and jaw misalignment in Germany are not available. Against this backdrop, the Sixth German Oral Health Study (DMS 6) included an orthodontic module. The following study objectives were pursued:

The primary objective of this study was to collect data on the prevalence of tooth and jaw misalignment in 8‑ and 9‑year-old children in Germany (primary endpoint).

The secondary objective of this study was to use this information to derive the need for orthodontic care provision (secondary endpoint).

## Short methodology overview

A detailed description of the scientific methodology of the Orthodontic Module of the Sixth German Oral Health Study can be found as an independent article in this special issue (Jordan et al. in this issue).

This short overview aims to provide only basic information relating to the applied methodology.

### Study design and setting

**Fig. 1:**
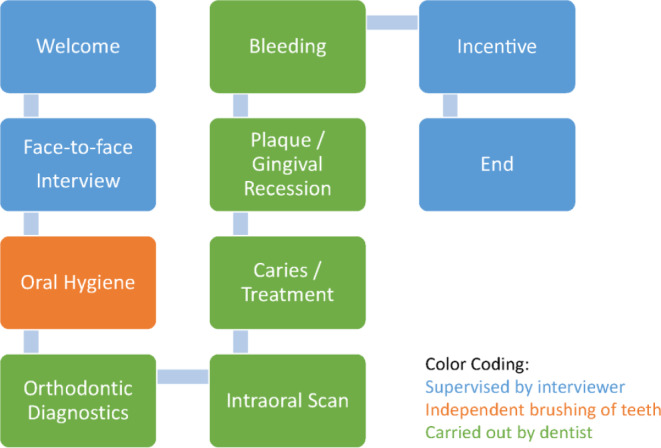
Process organization at the study center from the perspective of the study participants Organisation der Abläufe im Untersuchungszentrum aus Sicht der Studienteilnehmenden

This is an oral–epidemiological investigation and social scientific survey at the national level with a focus on tooth and jaw misalignment. The investigation took place between January and March 2021 at 16 study centers across Germany (Fig. 1).

### Study participants

After obtaining addresses from the municipal administrations responsible for study centers, 1892 people in the birth cohorts 2011 and 2012 were invited to participate in the study. A total of 714 underwent dental examination and socioscientific surveying. All relevant data were available for 705 of the study participants and these were included in the statistical analysis. The response rate was 40.6%. Subsequently, in order to gain insights into possible systemic differences between study participants and nonparticipants, a survey of nonrespondents was conducted. As the analysis did not show any differences between the study participants and the surveyed nonparticipants, it can be assumed that there is no distortion of the study results caused by the proportion of nonrespondents and the study results can be viewed as representative.

### Endpoints

The primary endpoint “Prevalence of Tooth and Jaw Misalignment” was operationalized as follows: Orthodontic Indication Group: KIG 1 vs. KIG 2 vs. KIG 3 vs. KIG 4 vs. KIG 5.

The secondary endpoint “Need for Orthodontic Treatment Provision” was based on statutory health care provider criteria and operationalized as follows: KIG 1–2 vs. KIG 3–5.

Furthermore, epidemiological–orthodontic indices were calculated for an international comparison, which will be published elsewhere in this special issue (Kirschneck et al. in this issue).

## Results

### Sample characterization

In total, 705 study participants were included in the data analysis. 51.4% of the study participants were male and 48.6% were female. The ratio of 8‑year-old children (49.4%) to 9‑year-old children (50.6%) was balanced. The result data were weighted to correspond to the population distribution in the principal regions in Germany: 22.2% of study participants came from rural areas, 32.9% from major urban centers, and 38.6% from metropolitan regions. In all, 90.8% of study participants reported good or very good health. In contrast, only 66.9% reported having good or very good oral health. 81.4% of study participants reported that they regularly attend dental check-ups. 9.2% reported only occasionally visiting the dentist. 7.4% reported only visiting a dentist if they have problems with their teeth. 2.0% have never visited a dentist. 8.4% of study participants were in early stage orthodontic treatment. On average, the study participants had 23.4 natural teeth, of which 10.4 were first dentition and 13.0 were permanent dentition teeth. 0.6 teeth were missing. On average, 0.9 teeth were erupting. 61.9% of study participants were caries-free, and 92.4% of permanent dentition was caries-free. An overview of the prevalence of habits, dyskinesia, and dysfunctions is depicted in Table 1.

**Table 1:** Distribution of habits, dyskinesias, and dysfunctions Verteilung von Habits, Dyskinesien und Dysfunktionen

		%	(95% CI)	n
Breathing pattern	Nasal breathing	98.7	(97.5–99.3)	683
	Mouth breathing	1.3	(0.7–2.5)	9
If mouth breathing: type	Habitual	80.5	(48.1–94.9)	7
	Anatomical	19.5	(5.1–51.9)	2
Swallowing pattern	Somatic	98.2	(97.0–99.0)	671
	Visceral	1.8	(1.0–3.0)	12
Lip seal	Competent	92.2	(89.9–93.9)	636
	Incompetent	3.1	(2.0–4.7)	21
	Potentially competent	4.7	(3.4–6.6)	33
Mentalis habit		18.0	(15.4–21.1)	125
Tongue dyskinesia: biting		0.4	(0.1–1.2)	3
Tongue dyskinesia: pressing		0.3	(0.1–1.1)	2
Lip dyskinesia: sucking		2.1	(1.2–3.4)	14
Lip dyskinesia: biting		2.4	(1.5–3.8)	16
Lip dyskinesia: pressing		0.2	(0.1–0.9)	2
Inner cheek dyskinesia: sucking		0.3	(0.1–1.1)	2
Inner cheek dyskinesia: biting		13.7	(11.3–16.5)	95
Forced bite		24.8	(21.6–28.2)	162
Sigmatism or speech disorder		21.9	(19.0–25.1)	154
Chewing problems		6.5	(4.9–8.6)	46
Biting fingernails		26.9	(23.8–30.3)	190
Sleep disorders/snoring		18.1	(15.4–21.1)	128
Sucking dyskinesia		6.1	(4.6–8.1)	43

Results of the weighted analysis, therefore rounding differences may occur

*CI* Confidence Interval

### Primary endpoint

For sociomedical reasons, for the German health care system, the results are primarily presented on the basis of orthodontic indication groups (Tables 2 and 3). When interpreting the results, it should be noted that study participants may have several tooth and jaw misalignments. If these multiple misalignments belong to different induction groups (e.g., one study participant had an edge-to-edge bite and crowding at the same time), both findings are counted and listed in the table. This means that the individual table rows always add up to 100% (subject to rounding differences), because only the most serious finding was counted for one and the same misalignment. However, this does not apply to the column or total summation due to possible double counting of study participants.

**Table 2:** Orthodontic indication group overview—frequency distribution Übersicht Kieferorthopädische Indikationsgruppen – Häufigkeitsverteilung

Indication groups	No findings	Grade 1	Grade 2	Grade 3	Grade 4	Grade 5	Total
	*n*						*n*
A—Cranial abnormalities	689 (99.6%)	–	–	–	–	3 (0.4%)	692
D—Distal bite malocclusion	–	72 (10.3%)	484 (69.2%)	–	115 (16.5%)	22 (3.2%)	698
M—Mesial bite malocclusion	671 (96.0%)	–	–	–	24 (3.4%)	4 (0.6%)	698
O—Vertical open bite malocclusion	653 (92.9%)^a^		32 (4.6%)	11 (1.6%)	0 (0.0%)	7 (1.0%)	703
T—Vertical deep bite malocclusion	39 (5.7%)	230 (33.4%)	353 (51.2%)	67 (9.8%)	–	–	689
B—Buccal/Lingual occlusion	701 (99.7%)	–	–	–	2 (0.3%)	–	704
K—End-to-end/Crossbite	644 (91.6%)	–	19 (2.7%)	3 (0.4%)	37 (5.3%)	–	704
E—Crowding	275 (39.1%)^a^		364 (51.7%)	59 (8.4%)	5 (0.7%)	–	704
P—Lack of space	474 (69.7%)	–	160 (23.5%)	21 (3.1%)	25 (3.6%)	–	679

Results of the weighted analysis, therefore, rounding differences are possible

^a^Differentiation of “no findings” and “grade 1” is not possible with the collected data; therefore, these categories are listed together. The indication groups U (hypodontia) and S (eruption disorders) were not assessed during this study as no X‑ray diagnostic were used

**Table 3:** Orthodontic Indication Group (Kieferorthopädische Indikationsgruppen, KIG) severity classification according to gender, region, and socioeconomic status KIG(Kieferorthopädische Indikationsgruppen)-Schweregradeinteilung nach Geschlecht, Region und sozioökonomischem Status

KIG		Grade 1	Grade 2	Grade 3	Grade 4	Grade 5	Total
		% (95% CI)					n
Total		2.5 (1.6–4.0)	57.0 (53.3–60.6)	10.0 (8.0–12.4)	25.5 (22.4–28.9)	5.0 (3.6–6.9)	705
Gender	Male	2.5 (1.3–4.6)	57.4 (52.3–62.4)	8.7 (6.2–12.0)	26.4 (22.1–31.2)	5.0 (3.2–7.8)	362
	Female	2.6 (1.4–4.9)	56.5 (51.2–61.7)	11.3 (8.4–15.1)	24.6 (20.3–29.4)	4.9 (3.1–7.8)	343
Region	Northern Germany	4.4 (2.0–9.5)	58.6 (49.9–66.8)	11.7 (7.2–18.4)	22.6 (16.2–30.6)	2.7 (1.0–7.2)	127
	Southern Germany	1.5 (0.5–4.2)	57.2 (50.4–63.8)	14.3 (10.2–19.7)	20.6 (15.6–26.6)	6.4 (3.8–10.7)	205
	Western Germany	1.9 (0.8–4.5)	56.9 (50.6–62.8)	6.0 (3.7–9.7)	29.9 (24.6–35.9)	5.3 (3.1–8.8)	249
	Eastern Germany	3.7 (1.5–8.7)	55.3 (46.5–63.7)	9.0 (5.1–15.3)	27.8 (20.6–36.2)	4.3 (1.9–9.4)	124
SES	Low	1.5 (0.4–5.6)	55.4 (46.6–63.9)	10.1 (5.9–16.6)	27.20 (20.2–35.7)	5.7 (2.8–11.3)	124
	Moderate	3.2 (1.9–5.6)	58.6 (53.6–63.6)	11.4 (8.5–15.0)	22.3 (18.3–26.8)	4.5 (2.8–7.1)	370
	High	2.6 (0.9–7.2)	55.1 (46.2–63.6)	5.8 (2.8–11.4)	36.6 (28.6–45.4)	0.0 (0.0–3.0)	122

Results of the weighted analysis, therefore, rounding differences may occur

*KIG* Kieferorthopädische Indikationsgruppen (Orthodontic Indication Groups), *SES* Socioeconomic status, *CI* confidence interval

The most frequent finding was distal bite (overbite; 88.9%). In contrast to the other indication groups, in this case, severity grade 1 (sagittal overjet of up to 3 mm) is still deemed a physiological dentition status, with pathological overbite enlargement being upwards of KIG grade 2. Also frequent were the indication groups crowding (at least 60.9%) and lack of space (30.3%). All other indication groups were each below 10%. Rare (< 1%) were buccal and lingual occlusions and craniofacial abnormalities. The prevalence of the indication groups hypodontia and eruption disorder could not be determined in this study due to the lack of radiological diagnostics. The most severe disease forms (KIG grade 5) were represented by distal bite (3.2%), open vertical overlap (open bite; 1.0%), mesial bite (0.6%), and craniofacial abnormalities (0.4%). With the exception of craniofacial abnormalities, which by definition can only occur as the most severe form of disease, the other most severe forms of disease were also observed in milder manifestations.

### Secondary endpoint

The need for orthodontic care provisions can be derived from the orthodontic indication group severity classifications. The following definitions were applied [14], resulting in the following percentages:

KIG grade 1: 2.5% of study participants were classified as KIG grade 1.

This also included the 0.7% of study participants who has no tooth misalignment and no orthodontic findings (eugnathic dentition). In these cases, there is absolutely no orthodontic treatment indicated. Classification as grade 1 can be justified solely by the fact that the physiological step in indication group D (sagittal overjet up to 3 mm) is defined as KIG grade 1.

A total of 1.8% of study participants displayed slight tooth misalignment and treatment may be desirable from an esthetic perspective, but not in the sense of a medical indication.

KIG grade 2: 57.0% of study participants had mild tooth misalignment that requires correction for medical reasons, but the cost of which will not be covered by the health insurance provider.

KIG grade 3: 10.0% of study participants had pronounced tooth misalignment that requires correction for medical reasons.

KIG grade 4: 25.5% of study participants had very pronounced tooth misalignment that requires treatment for medical reasons as soon as possible.

KIG grade 5: 5.0 of study participants had extremely pronounced tooth misalignment; it is imperative that they receive treatment for medical reasons.

The percentage of study participants requiring orthodontic treatment in accordance with the guidelines from the statutory health insurance providers is 40.4%. The percentage of study participants for whom, in principle, orthodontic treatment is indicated for medical reasons is 97.5%. Systemic differences in the need for care provision relating to gender, region, or social status were not observed. However, associations with the self-assessment of their own health status, habits, dyskinesias, and dysfunction arose. It was discovered that subjects requiring orthodontic treatment systematically rated their overall health and oral health status worse. Subjects requiring orthodontic treatment were more likely to systematically display mouth breathing (instead of nasal breathing), twice as likely to display incompetent lip sealing, and more likely to display other habits (mentalis habit, biting on their tongue, lip sucking, and fingernail biting), as well as sleep disorders and snoring.

*Craniofacial abnormalities* were rare. In this study, only 0.4% of study participants were diagnosed with this type of disease. All diagnosed cases were male.

*Hypodontia*, as described in the system to classify the need for orthodontic treatment, can only be definitively identified with the aid of X‑ray diagnostics. Therefore, orthodontic indication group U cannot be evaluated as part of DMS 6 because no X‑ray images are available. However, space maintainers (fixed) or replacement teeth (removable, e.g., child dentures) were clinically recorded. 0.4% of study participants had been fitted with a space maintainer following the loss of a tooth, and a further 0.2% had replacement teeth in the form of child dentures. For the reasons mentioned above, it is not possible to draw conclusions about the prevalence of indication group U based on this information.

*Tooth retention and tooth displacement*, as described in the KIG system to classify the need for orthodontic treatment, can only be definitively identified with the aid of X‑ray diagnostics. Therefore, orthodontic indication group S cannot be evaluated as part of DMS 6 because no X‑ray images are available. For this reason, a survey of these findings did not take place. An exception is ankylosis/partial retention of the 6‑year molars in the surveyed age group, which can be assessed without the aid of a radiological diagnostic scan. Despite the limitations, this parameter was recorded. None of the subjects displayed partial retention of the 6‑year molars, and 0.5% of study participants displayed partial retention affecting other permanent teeth (lateral incisors and second premolars). For the reasons mentioned above, it is not possible to draw conclusions about the prevalence of indication group S based on this information.

A *distal bite position malocclusion of the incisors* was frequent and affected 88.9% of study participants. Only 0.8% of study participants displayed no related findings. No tooth misalignment (sagittal overjet up to 3 mm, grade 1) was observed in 11.1% of subjects, and low-grade tooth misalignment (grade 2) was seen in the vast majority of study participants (69.2%). Systematic gender-related or regional differences were not observed. It is noticeable that distal bite cases requiring treatment were found more frequently in those with a higher social status.

In comparison with the distal findings, a *mesial bite position malocclusion of the incisors* was rather rare and affected only 4.0% of study participants; 96.0% of study participants displayed no related findings. All registered cases displayed pronounced (grade 4) or extremely pronounced (grade 5) tooth misalignment. Overbite was more prevalent among boys than girls. There were also differences in regional distribution. Overbite was more frequent in participants with a lower social status.

Discernible *vertical open* bite malocclusions were observed in 7.1% of study participants, while 92.9% of study participants displayed no related findings or low-grade findings. Less pronounced tooth misalignment (grade 2) was observed in 4.6% of participants, pronounced tooth misalignment (grade 3) in 1.6%, and extremely pronounced tooth misalignment (grade 5) in 1.0% of study participants. No systematic differences relating to gender, region, or social status were observed.

*Vertical deep bite* malocclusions were observed in 94.3% of the study participants. Only 5.7% of study participants displayed no related findings. Slight tooth misalignment (grade 1) was observed in one-third of participants and somewhat pronounced tooth misalignment (grade 2) in 51.2%. 9.8% of study participants displayed pronounced tooth misalignment with traumatic gingival contact (grade 3). No systematic differences relating to gender, region, or social status were observed.

*Transversal malocclusions in the form of buccal or lingual occlusions* were rare; they were observed in only 0.3% of study participants. All those affected displayed very pronounced tooth misalignment (grade 4). 99.7% of study participants displayed no related findings. No systematic differences relating to gender, region, or social status were observed.

Transversal malocclusions in the form of unilateral or bilateral crossbite were observed in 8.4% of study participants; 91.6% of study participants displayed no related findings. Somewhat pronounced tooth misalignment (grade 2) was observed in 2.7% of study participants in the form of end-to-end bite. Pronounced crossbite (grade 3) was observed in 0.4% of study participants and very pronounced crossbite (grade 4) in 5.3%. End-to-end bite and crossbite were more prevalent in girls. There were also differences in regional distribution. End-to-end bite and crossbite were more common in those of lower social status.

Discernible *vertical open bite malocclusions* were observed in 60.9% of study participants; 39.1% of study participants displayed no related findings or very low-grade findings. Somewhat pronounced tooth misalignment (grade 2) was displayed in 51.7% of study participants, pronounced tooth misalignment (grade 3) in 8.4%, and extremely pronounced tooth misalignment (grade 4) in 0.7%. No systematic differences relating to gender, region, or social status were observed.

*Lack of space* was observed in 30.3% of study participants; 69.7% of study participants displayed no related findings. Somewhat pronounced tooth misalignment (grade 2) was observed in 23.5% of study participants, 3.1% of study participants displayed pronounced (grade 3) findings, and 3.6% of study participants displayed extremely pronounced (grade 4) tooth misalignment. Lack of space was observed more frequently in boys than girls. There were also differences in regional distribution. No other differences related to social status were observed.

## Discussion

The need for care identified in this study in accordance with orthodontic indication groups (40.4%) generally corresponds to the figure of 41.1% from Glasl et al. in 2006 [10]. It can therefore be assumed that the need for orthodontic care in Germany has remained constant over the years. The percentage of study participants for whom, in principle, orthodontic treatment is indicated for medical reasons was 97.5%. This generally corresponds to earlier investigations, such as DMS 1, which reported the prevalence of absolute eugnathic dentition with no orthodontic abnormalities as 1%. In this study, the percentage of healthy natural orthodontic dentition was 0.7%.

### Strengths and limitations

A strength of DMS 6 is its representativeness regarding the population of 8‑ and 9‑year-old children in Germany, which was guaranteed via the geographical selection of one site in each federal state and the random sample collected from the municipal registration authorities. A limitation of this study is the fact that not all orthodontic abnormalities could be recorded: The KIG categories U (hypodontia) and S (eruption disorders, retention, and displacement) could not be assessed because, for ethical reasons, no radiological images of the study participants’ jaws could be taken. Due to the fact that, for the aforementioned reasons, the prevalence of KIG grades 3–5, which imply the need for orthodontic provision in KIG categories U and S, could not be surveyed, it can be assumed that the actual need for orthodontic care provision in the study population of 8‑ to 9‑year-old children is higher than the 40.4% identified during this study. Studies have shown that a prevalence of hypodontia in category U of approximately 5% and prevalence of retained/displaced tooth of approximately 6% must be assumed. A further limitation of the methodological aspect of this study is the application of orthodontic indication groups (KIG) as an epidemiological index for a population of 8‑ and 9‑year-old children, as this was developed to determine the extent of reimbursable orthodontic services in the context of statutory health insurance coverage for a population of over 10-year-olds. Therefore, there is a risk that the actual prevalence and the need for orthodontic care provision are underestimated, which will then manifest 1–2 years later in the studied population group as it is known that the majority of orthodontic abnormalities display an increase in prevalence during growth [11]. However, the selection of a collective of 8‑ and 9‑year-old children for DMS 6 was a conscious decision to avoid the possible disruptive influences of early orthodontic treatment which is often administered before 10 years of age.

### Interpretation

Regarding the geographical distribution of the individual prevalences and KIG severity grading, it is noticeable that there are no significant differences between the subpopulations of northern, southern, eastern, or western Germany. The higher grades of the KIG categories M (sagittal discrepancy negative overjet) and K (transversal abnormalities) are an exception as they tend to be more frequent in southern and eastern Germany but underrepresented in northern Germany. In contrast, KIG category D (sagittal discrepancy increased overjet) appears to be more frequent in northern Germany than in southern and eastern Germany. There were also no significant differences observed in the individual prevalences and KIG severity classification relating to socioeconomic status (SES). Existing differences can almost certainly be attributed to the sample effects relating to the limited number of cases included in the study.

### Future research impulses

During DMS 7, the study participants of the orthodontic module in DMS 6 should be examined again with the aim of obtaining, for the first time, longitudinal data related to the development of orthodontic abnormalities with and without orthodontic treatment having been carried out in the meantime. In some cases, the efficacy of orthodontic therapeutic procedures can also be evaluated. In future epidemiological studies, more attention should be placed on the reliable surveying of myofunctional habits and dyskinesias, as these represented a significant exogenous etiological factor for the onset of orthodontic abnormalities [15].

## Conclusion

To determine tooth and jaw misalignment, this study applies the German orthodontic indication groups along with internationally established orthodontic–epidemiological indices to the sample group of 8‑ and 9‑year-old children (early mixed dentition). The primary aim was to determine the need for orthodontic treatment provision in a group with a large proportion of untreated patients. A need for orthodontic treatment provision was identified in 40.4% of subjects. However, it must be taken into account that in later stage mixed dentition (main treatment period in accordance with statutory health care provider guidelines), an increase can be expected due to the progression of tooth and jaw misalignment, and therefore the Orthodontic Indication Group [Kieferorthopädische Indikationsgruppen, KIG] categories U (hypodontia) and S (eruption disorders, retention, and displacement) could not be taken into account. When applying the corresponding guidelines, in international comparison, neither an underprovision nor an overprovision of treatment in Germany is observed. A comparison with the invoicing data of the National Association of Health Insurance Dentists (Kassenzahnärztliche Bundesvereinigung, KZBV) also shows that the need for orthodontic treatment provision generally corresponds to the actual provision of treatment.

## Supplementary Information

German Version
