# Supplementary material for: Prevalence of malocclusions in 8- and 9-year-old children in Germany—Results of the Sixth German Oral Health Study (DMS 6)
Source: J Orofac Orthop. 2023 Feb 1;84(Suppl 1):1–9. doi: 10.1007/s00056-022-00437-z (PMC9892134; doi:10.1007/s00056-022-00437-z)
Supplement: Supplementary file 1 — German Version [file 56_2022_437_MOESM1_ESM.pdf]

Verbreitung von Zahn- und Kieferfehlstellungen 8- und 9-jähriger Kinder in Deutschland – Ergebnisse der Sechsten Deutschen Mundgesundheitsstudie (DMS • 6)

Kurztitel: Prävalenzen von Zahn- und Kieferfehlstellungen bei 8- und 9-Jährigen in Deutschland

Andreas Rainer Jordan[1*], Katrin Kuhr[1], Nicolas Frenzel Baudisch[1], Christian Kirschneck[2]

[1] Institut der Deutschen Zahnärzte (IDZ), Universitätsstr. 73, 50931 Köln, Deutschland

[2] Poliklinik für Kieferorthopädie, Universitätsklinikum Regensburg, Franz-Josef-Strauß-Allee 11, 93053 Regensburg, Deutschland

[*]Korrespondenzautor:
Prof. Dr. A. Rainer Jordan
r.jordan@idz.intitute

Finanzierung. Die Studie wurde finanziert von der Deutschen Gesellschaft für Kieferorthopädie e.V.

Erklärungen

Interessenskonflikte. A.R. Jordan, K. Kuhr und N. Frenzel Baudisch erklären, dass sie keine finanziellen oder nicht-finanziellen Interessen haben, die direkt oder indirekt mit der zur Veröffentlichung eingereichten Arbeit zusammenhängen. C. Kirschnek erklärt den Erhalt von Honorar als wissenschaftlicher Berater der Sechsten Deutschen Mundgesundheitsstudie.

Ethikvotum. Die Ethikkommission der Universität Witten/Herdecke hat die Studie vorab unter ethischen Gesichtspunkten geprüft und ihr zugestimmt (Nr. 113/2020). Die Studie wurde erst nach Vorliegen einer zustimmenden Bewertung der zuständigen Ethikkommission begonnen.

Einverständnis zur Teilnahme: Die schriftliche Einwilligung wurde von den Patienten oder bei Kindern unter 18 Jahren von ihren Eltern bzw. ihren gesetzlichen Vertretern eingeholt.

Studienregistrierung. Vor Beginn wurde die Registrierung der Studie beim Deutschen Register Klinische Studien veranlasst (DRKS: www.drks.de): DRKS00022472**.**

Zusammenfassung

Ziel Aktuelle, bevölkerungsweite Daten zur Verbreitung von Zahn- und Kieferfehlstellungen in Deutschland liegen nicht vor. Es war daher das primäre Ziel dieser Studie, die Verbreitung von Zahn- und Kieferfehlstellungen bei 8/9-jährigen Kindern in Deutschland zu erfassen. Es war das sekundäre Ziel dieser Studie, daraus den kieferorthopädischen Versorgungsbedarf abzuleiten.

Methode Es handelt sich um einen oralepidemiologischen Untersuchungs- und sozialwissenschaftlichen Befragungssurvey auf national repräsentativer Ebene mit Scherpunkt auf Zahn- und Kieferfehlstellungen. Die Untersuchungen fanden von Januar bis März 2021 in 16 Studienzentren in Deutschland statt. Für 705 Studienteilnehmende lagen alle relevanten Daten vor und sie wurden in die statistische Auswertung einbezogen.

Ergebnisse Am häufigsten kamen mit 88,9 % Überbisse vor. Ebenfalls weit verbreitet waren Engstand mit mindestens 60,9 % sowie Platzmangel mit einem Anteil von 30,3 %. Alle anderen Indikationsgruppen wiesen jeweils einen Anteil von unter 10 % auf. Selten (<1 %) wurden Bukkal-/Lingualokklusionen sowie kraniofaziale Anomalien vorgefunden. Die schwerwiegendsten Erkrankungsformen (KIG Grad 5) stellten mit 3,2 % der Überbiss, mit 1,0 % der offene Biss, mit 0,6 % der Vorbiss und die kraniofazialen Anomalien (0,4 %) dar. Der Anteil der Studienteilnehmenden, bei denen nach den Richtlinien der gesetzlichen Krankenversicherung eine kieferorthopädische Behandlung angezeigt ist, lag bei 40,4 %. Der Anteil der Studienteilnehmenden, bei denen aus medizinischen Gründen eine kieferorthopädische Behandlung grundsätzlich angezeigt sein kann, lag bei insgesamt 97,5 %. Systematische Unterschiede im Hinblick auf das Geschlecht, die Region oder den Sozialstatus wurden beim Versorgungsbedarf nicht festgestellt.

Schlussfolgerungen Der im Rahmen dieser Studie nach kieferorthopädischen Indikationsgruppen ermittelte Versorgungsbedarf deckt sich weitgehend mit früheren Untersuchungen. Damit liegt nahe, dass der kieferorthopädische Behandlungsbedarf in Deutschland über die Jahre weitgehend konstant geblieben ist.

Schlüsselwörter ICON, Epidemiologie, Kieferorthopädie, KIG, Versorgungsforschung

Einleitung

Zahn- und Kieferfehlstellungen gehören neben Karies und Parodontalerkrankungen zu den häufigsten Gesundheitsbeeinträchtigungen der Mundhöhle [1]. Erkrankungen des Kauorgans, also der Zähne, Kiefer, Kiefergelenke und Kaumuskulatur, können das Wohlbefinden und die Lebensqualität gravierend beeinträchtigen, indem sie Schmerzen und Leid verursachen, die Nahrungsaufnahme bzw. Nahrungswahl beeinflussen oder das Sprechen erschweren [2]. In diesem Sinne ist die Kieferorthopädie eine stark präventiv ausgerichtete Disziplin, wenn durch eine kieferorthopädische Behandlung Folgeerkrankungen verhindert werden können. So ist bekannt, dass kieferorthopädische Anomalien mit Einschränkungen der Kaufunktion [3], der Atmung [3, 4], der Phonetik und des Schluckens [5, 6] assoziiert sind und auch eine vergrößerte sagittale Frontzahnstufe das Risiko signifikant erhöht, ein Frontzahntrauma zu erleiden [7] bzw. eine kieferorthopädische Korrektur der Stufe dieses Risiko wirksam senken kann [8]. Die Ursachen kieferorthopädischer Erkrankungen sind multifaktoriell und reichen von genetischen, epigenetischen und funktionellen bis hin zu umweltbedingten Faktoren. Auch der Ausprägungsgrad der einzelnen Erkrankungen ist ausgesprochen variabel. Dementsprechend umfangreich sind die Therapieoptionen. Genetische und epigenetische Faktoren lassen sich durch eine kieferorthopädische Therapie nur schwer beeinflussen; eine Behandlung richtet sich vornehmlich gegen die Folgen. Bei funktionellen und umweltbedingten Faktoren hingegen bestehen grundsätzlich Präventionsmöglichkeiten und oftmals eine kausale Therapieoption. Traditionell wurden Zahn- und Kieferfehlstellungen nach der Okklusionsbeziehung der Sechsjahrmolaren eingeteilt, den sogenannten Angle-Klassen, und daraus Rückschlüsse auf die Lagebeziehung der Kiefer zueinander gezogen. Die Verbreitung der Angle-Klassen ist regional sehr unterschiedlich, wobei global sämtliche Angle-Klassen vertreten sind [1]: Im bleibenden Gebiss herrscht global betrachtet die Verbreitung der Angle-Klasse I mit einem Anteil von etwa 75 % vor, gefolgt von Erkrankungen der Angle-Klasse II mit etwa 20 %. Die Angle-Klasse III kommt zu etwa 6 % vor. In einer kieferorthopädisch-epidemiologischen Untersuchung an 494 9-jährigen Schulkindern im Südwesten Deutschlands wurde ebenfalls festgestellt, dass die Angle-Klasse I bei den Kindern am häufigsten vorkam, gefolgt von den Angle-Klassen II und III [9]. Angle-Klasse-II-Verzahnungen wurden in dieser Studie zu etwa 20 % gefunden; der Wert liegt damit im Varianzbereich der bei Alhammadi et al. (2018) [1] berichteten Prävalenzen. Angle-Klasse III kam bei 3 % der Untersuchten vor. Eine epidemiologisch-kieferorthopädische Untersuchung im Rahmen der schulzahnärztlichen Untersuchung in Frankfurt am Main bei 1.251 Schülern im Alter von etwa 9–11 Jahren hat die kieferorthopädischen Befunde nach dem Befundschema der gesetzlichen Krankenversicherung

in Deutschland ausgewertet, den Kieferorthopädischen Indikationsgruppen (KIG) [10]. Nach dieser Studie fand sich eine Behandlungsindikation im Sinne der gesetzlichen Krankenversicherung (KIG >= 3) in 41,4 % aller untersuchten Fälle. Stahl et al. haben herausgefunden, dass Habits, Dysfunktionen und Dyskinesien vom Milch- zum Wechselgebiss signifikant ansteigen [11]. Dabei wurden orale Habits von Mädchen häufiger gezeigt als von Jungen, Artikulationsstörungen hingegen traten häufiger bei Jungen auf. Insgesamt stellte sich heraus, dass myofunktionelle Störungen häufiger bei Kindern mit vergrößerter sagittaler Stufe, bei offenem Biss, seitlichem Kreuzbiss und bei Progenie (Angle-Klasse III) gefunden wurden. In einem weiteren Bericht desselben Autorenteams wurden im Milchgebiss physiologische okklusale Lagebeziehungen bei einem Viertel der Kinder gefunden; der Anteil sank deutlich auf 7 %, sobald Kinder im Wechselgebiss untersucht wurden [12].

Das Institut der Deutschen Zahnärzte (IDZ) hat im Jahr 1989 mit der ersten Deutschen Mundgesundheitsstudie (DMS I) den Grundstein für ein bevölkerungsrepräsentatives sozial-epidemiologisches Monitoring der Mundgesundheit und zahnmedizinischen Versorgung in der Bundesrepublik Deutschland geschaffen [13]. Zahn- und Kieferfehlstellungen wurden bislang alleinig im Rahmen der ersten Deutschen Mundgesundheitsstudie im Jahr 1989 in den alten Bundesländern untersucht. Aktuelle, bevölkerungsweite Daten zur Verbreitung von Zahn- und Kieferfehlstellungen in Deutschland liegen nicht vor. Vor diesem Hintergrund wurde die Sechste Deutsche Mundgesundheitsstudie (DMS • 6) um ein kieferorthopädisches Modul erweitert. Darin wurden folgende Studienziele verfolgt:

1. Es war das primäre Ziel dieser Studie, die Verbreitung von Zahn- und Kieferfehlstellungen bei 8/9-jährigen Kindern in Deutschland zu erfassen (primäre Zielgröße).
2. Es war das sekundäre Ziel dieser Studie, daraus den kieferorthopädischen Versorgungsbedarf abzuleiten (sekundäre Zielgröße).

Kurz-Methodik

Eine ausführliche Darstellung der wissenschaftlichen Methodik des kieferorthopädischen Moduls der Sechsten Deutschen Mundgesundheitsstudie findet sich als eigenständiger Artikel in diesem Sonderband des *Journal of Orofacial Orthopedics* (Jordan et al.). An dieser Stelle soll lediglich auf methodische Eckdaten verwiesen werden.

Studiendesign und Setting

Es handelt sich um einen oralepidemiologischen Untersuchungs- und sozialwissenschaftlichen Befragungssurvey auf national repräsentativer Ebene mit Scherpunkt auf Zahn- und Kieferfehlstellungen. Die Untersuchungen fanden von Januar bis März 2021 in 16 Studienzentren in Deutschland statt (**Abb. 1**).

Studienteilnehmende

Nach einer Adressziehung in den Gemeindeverwaltungen der Studienzentren wurden 1.892 Personen der Geburtskohorten 2011 und 2012 zur Teilnahme an der Studie eingeladen. Insgesamt wurden 714 Studienteilnehmende zahnmedizinisch untersucht und sozialwissenschaftlich befragt. Für 705 Studienteilnehmende lagen alle relevanten Daten vor und sie wurden in die statistische Auswertung einbezogen. Die Response-Rate lag bei 40,6 %. 51,4 % der Studienteilnehmenden waren männlichen Geschlechts (weiblich: 48,6 %), der Anteil der 8-Jährigen lag bei 49,4 % (9-Jährige: 50,6 %). Um Erkenntnisse über etwaige systematische Unterschiede zwischen Studienteilnehmenden und Nicht-Studienteilnehmenden zu gewinnen, wurde anschließend eine Nonresponder-Befragung durchgeführt. Da die Analyse keine systematischen Unterschiede zwischen den Studienteilnehmenden und den befragten Nicht-Studienteilnehmenden aufzeigte, ist von keiner Verzerrung der Studienergebnisse durch den Anteil der Nonresponder auszugehen und die Studienergebnisse können als repräsentativ angesehen werden.

Zielgrößen

Die primäre Zielgröße „Verbreitung von Zahn- und Kieferfehlstellung“ wurde wie folgt operationalisiert: Kieferorthopädische Indikationsgruppe: KIG 1 vs. KIG 2 vs. KIG 3 vs. KIG 4 vs. KIG 5.

Die sekundäre Zielgröße „Kieferorthopädischer Versorgungsbedarf“ wurde zum einen basierend auf den GKV-Kriterien wie folgt operationalisiert: KIG 1–2 vs. KIG 3–5.

Weitere epidemiologisch-kieferorthopädische Indizes wurden für einen internationalen Vergleich berechnet., die andernorts in diesem Sonderband des *Journal of Orofacial Orthopedics* veröffentlicht werden (Kirschneck et al.).

Ergebnisse

Charakterisierung der Stichprobe

Insgesamt wurden 705 Studienteilnehmende in die Datenauswertung eingeschlossen. 51,4 % dieser Studienteilnehmenden waren männlichen Geschlechts, 48,6 % weiblich. Das Verhältnis von 8-jährigen Kindern (49,4 %) zu 9-jährigen Kindern (50,6 %) war ausgewogen. Die Ergebnisdaten wurden so gewichtet, dass sie der Bevölkerungsverteilung der kardinalen Großregionen in Deutschland entsprachen. 22,2 % der Studienteilnehmenden kamen aus ländlichen Regionen, 39,2 % aus städtischen sowie 38,6 % aus Großstadtregionen. 90,8 % der Studienteilnehmenden gaben an, über einen guten oder sehr guten allgemeinen Gesundheitszustand zu verfügen. Dahingegen gaben lediglich 66,9 % der Studienteilnehmenden an, einen guten oder sehr guten Mundgesundheitszustand aufzuweisen. 81,4 % der Studienteilnehmenden gaben an, regelmäßig zur Kontrolle zum Zahnarzt zu gehen, weitere 9,2 % gaben gelegentliche Zahnarztbesuche an. 7,4 % haben angegeben, den Zahnarzt oder die Zahnärztin nur bei Problemen mit den Zähnen aufzusuchen. 2,0 % sind noch nie beim Zahnarzt gewesen. Es ergab sich ein Anteil von 8,4 % der Studienteilnehmenden in kieferorthopädischer Frühbehandlung. Durchschnittlich wiesen die Studienteilnehmenden 23,4 eigene Zähne auf, davon waren 10,4 Zähne der ersten Dentition und 13,0 Zähne gehörten zum permanenten Gebiss, 0,6 Zähne fehlten. Durchschnittlich befanden sich 0,9 Zähne im Durchbruch. Kariesfrei waren 61,9 % der Studienteilnehmenden, bezogen auf das permanente Gebiss waren 92,4 % kariesfrei.

Eine Übersicht über die Verbreitung von Habits, Dyskinesien und Dysfunktionen ist in **Tab. 1** dargestellt.

Primäre Zielgröße

Die Ergebnisdarstellung erfolgt aus sozialmedizinischen Gründen für das deutsche Gesundheitssystem vornehmlich anhand Kieferorthopädischer Indikationsgruppen (**Tab. 2**, **Tab. 3**). Bei der Interpretation der Ergebnisse ist zu beachten, dass ein Studienteilnehmender grundsätzlich mehrere Zahn- und Kieferfehlstellungen gleichzeitig aufweisen kann. Bezogen sich diese multiplen Fehlstellungen auch auf unterschiedliche Indikationsgruppen (ein Studienteilnehmender wies z. B. einen Kopfbiss und gleichzeitig einen Engstand auf), wurden beide Befunde gezählt und in die Tabelle eingetragen. Das bedeutet, dass die einzelnen Zeilen in der Tabelle zwar immer auf 100 % aufgehen (vorbehaltlich Rundungsdifferenzen), denn für ein und dieselbe Fehlstellung wurde immer nur der schwerwiegendste Befund gezählt. Dies gilt

jedoch wegen möglicher Doppelzählungen von Studienteilnehmenden nicht für die Spalten- und Gesamtsummation!

Am häufigsten kamen mit 88,9 % sagittale Stufen distal (Überbiss) vor. Anders als bei den anderen Indikationsgruppen gibt hier der Schweregrad 1 (sagittale Frontzahnstufe von bis zu 3 mm) eine noch physiologische Gebisssitution an, pathologisch vergrößerte Frontzahnstufen liegen erst ab KIG Grad 2 vor. Ebenfalls weit verbreitet waren die Indikationsgruppen Engstand mit mindestens 60,9 % sowie Platzmangel mit einem Anteil von 30,3 %. Alle anderen Indikationsgruppen wiesen jeweils einen Anteil von unter 10 % auf. Selten (<1 %) wurden Bukkal-/Lingualokklusionen sowie kraniofaziale Anomalien vorgefunden. Die beiden Indikationsgruppen Zahnunterzahl und Durchbruchsstörungen konnten im Rahmen der vorliegenden Studie mangels radiologischer Diagnostik nicht bestimmt werden. Die schwerwiegendsten Erkrankungsformen (KIG Grad 5) stellten mit 3,2 % die sagittale Stufe distal (Überbiss), mit 1,0 % die vertikale Stufe offen (offener Biss), mit 0,6 % die sagittale Stufe mesial (Vorbiss) und die kraniofazialen Anomalien (0,4 %) dar. Mit Ausnahme der kraniofazialen Anomalien, die definitionsgemäß nur als schwerwiegendste Erkrankungsform vorkommen können, zeigten sich die anderen schwerwiegendsten Erkrankungsformen auch in milderen Erscheinungsformen.

Sekundäre Zielgröße

Der kieferorthopädische Versorgungsbedarf kann aus der Schweregradeinteilung der Kieferorthopädischen Indikationsgruppen abgeleitet werden. Hierbei wurde folgende Definition zugrunde gelegt [14] und es ergaben sich folgende Anteile:

- KIG Grad 1: 2,5 % der Studienteilnehmenden wurde der KIG Grad 1 zugewiesen.

Dazu zählten auch 0,7 % der Studienteilnehmenden, die keine Zahnfehlstellungen aufwiesen und ohne kieferorthopädischen Befund (eugnathes Gebiss) waren. In diesen Fällen liegt absolut keine Behandlungsindikation für eine kieferorthopädische Therapie vor. Die Zuordnung zu Grad 1 ist alleine dadurch begründet, dass in der Indikationsgruppe D die physiologische Stufe (sagittale Frontzahnstufe von bis zu 3 mm) als KIG Grad 1 definiert ist.

1,8 % der Studienteilnehmenden wiesen leichte Zahnfehlstellungen auf, deren Behandlung aus ästhetischen Gründen wünschenswert sein kann, jedoch nicht zu Lasten der Krankenkassen.

- KIG Grad 2: 57,0 % der Studienteilnehmenden wiesen Zahnfehlstellungen geringer Ausprägung auf, die zwar aus medizinischen Gründen eine Korrektur erforderlich machen, deren Kosten jedoch nicht von den Krankenkassen übernommen werden.
- KIG Grad 3: 10,0 % der Studienteilnehmenden wiesen ausgeprägte Zahnfehlstellungen auf, die aus medizinischen Gründen eine Behandlung erforderlich machen.
- KIG Grad 4: 25,5 % der Studienteilnehmenden wiesen stark ausgeprägte Zahnfehlstellungen auf, die aus medizinischen Gründen dringend eine Behandlung erforderlich machen.
- KIG Grad 5: 5,0 % der Studienteilnehmenden wiesen extrem stark ausgeprägte Zahnfehlstellungen auf, die aus medizinischen Gründen unbedingt eine Behandlung erforderlich machen.

Der Anteil der Studienteilnehmenden, bei denen nach den Richtlinien der gesetzlichen Krankenversicherung eine kieferorthopädische Behandlung angezeigt ist, lag bei 40,4 %. Der Anteil der Studienteilnehmenden, bei denen aus medizinischen Gründen eine kieferorthopädische Behandlung grundsätzlich angezeigt sein kann, lag bei insgesamt 97,5 %. Systematische Unterschiede im Hinblick auf das Geschlecht, die Region oder den Sozialstatus wurden beim Versorgungsbedarf nicht festgestellt. Allerdings stellten sich Assoziationen zur Selbsteinschätzung des eigenen Gesundheitszustandes, zu Habits, Dyskinesien und Dysfunktionen dar. Es stellte sich heraus, dass Probanden, die einen kieferorthopädischen Versorgungsbedarf aufwiesen, ihren allgemeinen Gesundheitszustand wie auch ihren Mundgesundheitszustand systematisch schlechter einstuften. Probanden mit kieferorthopädischem Versorgungsbedarf zeigten systematisch häufiger eine Mundatmung (statt einer Nasenatmung), zeigten etwa doppelt so häufig einen inkompetenten Lippenschluss und häufiger weitere Angewohnheiten (Mentalishabit, Zungenbeißen, Lippensaugen, Fingernägelkauen) sowie Schlafstörungen und Schnarchen.

Kraniofaziale Anomalien kamen selten vor. In der vorliegenden Untersuchung wurden 0,4 % der Studienteilnehmenden mit derartigen Erkrankungen diagnostiziert. Alle diagnostizierten Fälle waren männlichen Geschlechts.

Hypodontien, wie sie in dem Schema zur Einstufung des kieferorthopädischen Behandlungsbedarfs beschrieben werden, können nur mithilfe strahleninvasiver Verfahren sicher erkannt werden. Die Kieferorthopädische Indikationsgruppe U kann daher im Rahmen der DMS • 6 nicht beurteilt werden, da keine Röntgenbilder vorliegen. Es wurde allerdings

klinisch erfasst, ob ein Lückenhalter (festsitzend) oder ein ersetzter Zahn (abnehmbar, z. B. Kinderprothese) vorhanden ist. Bei 0,4 % der Studienteilnehmenden war infolge Zahnverlustes ein Lückenhalter inseriert und weitere 0,2 % der Studienteilnehmenden wiesen ersetzte Zähne im Sinne einer Kinderprothese auf. Rückschlüsse auf Prävalenzen der Indikationsgruppe U sind basierend auf diesen Angaben aus oben genannten Gründen nicht möglich.

Zahnretentionen und Zahnverlagerungen, wie sie in dem Schema zur Einstufung des kieferorthopädischen Behandlungsbedarfs anhand KIG erfasst werden, können nur mithilfe strahleninvasiver Verfahren sicher erkannt werden. Die Kieferorthopädische Indikationsgruppe S kann daher im Rahmen der DMS • 6 nicht beurteilt werden, da keine Röntgenbilder vorliegen. Aus diesem Grund findet eine Erhebung der genannten Befunde grundsätzlich nicht statt. Eine Ausnahme, die in der untersuchten Altersgruppe ohne radiologische Diagnostik mithilfe der Scans erfasst werden kann, betrifft die Ankylose/Halbretention der Sechsjahrmolaren. Dieser Parameter wird trotz der genannten Einschränkungen erfasst. Bei keinem der Probanden stand ein Sechsjahrmolar in Halbretention, bei 0,5 % der Studienteilnehmenden standen sonstige bleibende Zähne in Halbretention (2er, 5er). Rückschlüsse auf Prävalenzen der Indikationsgruppe S sind basierend auf diesen Angaben aus oben genannten Gründen nicht möglich.

Abweichungen in der sagittalen Stufe der Schneidezähne *distal* waren weit verbreitet und betrafen 88,9 % der Studienteilnehmenden. Lediglich 0,8 % der Studienteilnehmenden wiesen diesbezüglich keinen Befund auf. Keine Zahnfehlstellungen (sagittale Frontzahnstufe von bis zu 3 mm, Grad 1) wurden bei 11,1 % festgestellt und Zahnfehlstellungen geringer Ausprägung (Grad 2) zeigten sich bei der überwiegenden Mehrheit der Studienteilnehmenden (69,2 %). Systematische geschlechtsbezogene oder regionale Unterschiede wurden nicht festgestellt. Auffällig ist, dass behandlungsbedürftige sagittale Stufen distal vermehrt bei einem hohen Sozialstatus festgestellt wurden.

Abweichungen in der sagittalen Stufe mesial der Schneidezähne waren im Vergleich zu den Abweichungen distal eher selten und betrafen 4,0 % der Studienteilnehmenden. 96,0 % der Studienteilnehmenden zeigten diesbezüglich keinen Befund. Alle registrierten Befunde wiesen stark (Grad 4) oder extrem stark (Grad 5) ausgeprägte Zahnfehlstellungen auf. Jungen wiesen systematisch mehr Vorbisse auf als Mädchen. Es stellten sich ebenfalls Unterschiede in der Regionalverteilung dar. Studienteilnehmende mit niedrigem Sozialstatus wiesen häufiger Vorbisse auf.

Messbare Abweichungen in der *vertikalen Stufe offen* (offener Biss) lagen bei 7,1 % der Studienteilnehmenden vor. 92,9 % der Studienteilnehmenden wiesen diesbezüglich keinen oder nur einen geringgradigen Befund auf. Zahnfehlstellungen geringer Ausprägung (Grad 2) lagen bei 4,6 %, ausgeprägte Zahnfehlstellungen (Grad 3) bei 1,6 % und extrem stark ausgeprägte Zahnfehlstellungen (Grad 5) bei 1,0 % der Studienteilnehmenden vor. Systematische Unterschiede im Hinblick auf das Geschlecht, die Region oder den Sozialstatus wurden nicht festgestellt.

Abweichungen in der *vertikalen Stufe tief* (tiefer Biss) lagen bei 94,3 % der Studienteilnehmenden vor. Lediglich 5,7 % der Studienteilnehmenden wiesen diesbezüglich keinen Befund auf. Leichte Zahnfehlstellungen (Grad 1) lagen bei einem Drittel und Zahnfehlstellungen geringer Ausprägung (Grad 2) bei 51,2 % vor. 9,8 % der Studienteilnehmenden zeigten ausgeprägte Zahnfehlstellungen mit traumatischem Gingivakontakt (Grad 3). Systematische Unterschiede im Hinblick auf das Geschlecht, die Region oder den Sozialstatus wurden nicht festgestellt.

Transversale Abweichungen im Sinne einer Bukkal-/Lingualokklusion kamen selten vor; sie wurden bei 0,3 % der Studienteilnehmenden festgestellt. Sie wiesen alle eine stark ausgeprägte Zahnfehlstellung (Grad 4) auf. 99,7 % der Studienteilnehmenden wiesen diesbezüglich keinen Befund auf. Systematische Unterschiede im Hinblick auf das Geschlecht, die Region oder den Sozialstatus wurden nicht festgestellt.

Transversale Abweichungen im Sinne eines beid- oder einseitigen Kreuzbisses kamen bei 8,4 % der Studienteilnehmenden vor. 91,6 % der Studienteilnehmenden wiesen diesbezüglich keinen Befund auf. Zahnfehlstellungen geringer Ausprägung (Grad 2) lagen bei 2,7 % der Studienteilnehmenden vor. Hierbei handelte es sich um Kopfbisse. Ausgeprägte Kreuzbisse (Grad 3) wurden bei 0,4 % der Studienteilnehmenden gefunden und stark ausgeprägte Kreuzbisse (Grad 4) bei 5,3 % der Studienteilnehmenden. Mädchen wiesen mehr Kopfbisse/Kreuzbisse auf als Jungen. Es stellten sich ebenfalls Unterschiede in der Regionalverteilung dar. Studienteilnehmende mit niedrigem Sozialstatus wiesen häufiger Kopfbisse/Kreuzbisse auf.

Messbare *Abweichungen in der vertikalen Stufe offen* (offener Biss) lagen bei 60,9 % der Studienteilnehmenden vor. 39,1 % der Studienteilnehmenden wiesen diesbezüglich keinen oder nur einen leichten Befund auf. Zahnfehlstellungen geringer Ausprägung (Grad 2) lagen bei 51,7 %, ausgeprägte Zahnfehlstellungen (Grad 3) bei 8,4 % und extrem stark ausgeprägte Zahnfehlstellungen (Grad 4) bei 0,7 % der Studienteilnehmenden vor. Systematische

Unterschiede im Hinblick auf das Geschlecht, die Region oder den Sozialstatus wurden nicht festgestellt.

Platzmangel kam bei 30,3 % der Studienteilnehmenden vor. 69,7 % der Studienteilnehmenden wiesen diesbezüglich keinen Befund auf. Zahnfehlstellungen geringer Ausprägung (Grad 2) lagen bei 23,5 % der Studienteilnehmenden vor. 3,1 % der Studienteilnehmenden wiesen ausgeprägte (Grad 3) und 3,6 % der Studienteilnehmenden extrem stark ausgeprägte (Grad 4) Zahnfehlstellungen auf. Jungen wiesen häufiger Platzmangel auf als Mädchen. Es stellten sich ebenfalls systematische Unterschiede in der Regionalverteilung dar. Weitere systematische Unterschiede im Hinblick auf den Sozialstatus wurden nicht festgestellt.

Diskussion

Der im Rahmen dieser Studie nach Kieferorthopädischen Indikationsgruppen ermittelte Versorgungsbedarf (40,4 %) deckt sich weitgehend mit dem von Glasl et al. im Jahre 2006 [10], den die Autoren mit 41,4 % bezifferten. Damit liegt nahe, dass der kieferorthopädische Behandlungsbedarf in Deutschland über die Jahre weitgehend konstant geblieben ist. Der Anteil der Studienteilnehmenden, bei denen aus medizinischen Gründen eine kieferorthopädische Behandlung grundsätzlich angezeigt sein kann, lag bei insgesamt 97,5 %. Dies deckt sich weitgehend mit früheren Untersuchungen wie der DMS I, nach denen die Prävalenz eines absolut eugnathen Gebisses ohne kieferorthopädische Anomalien mit 1 % angegeben wurde. In der vorliegenden Studie lag der Anteil kieferorthopädisch-naturgesunder Gebisse bei 0,7 %.

Stärken und Schwächen

Eine Stärke der DMS • 6 stellt die Repräsentativität bezüglich der Bevölkerung der 8- und 9-Jährigen in Deutschland dar, welche durch die geografische Berücksichtigung von je einem Standort pro Bundesland sowie der zufälligen Stichprobenziehung via Einwohnermeldeämter gewährleistet wurde. Eine Limitation der Studie ist darin zu sehen, dass nicht alle kieferorthopädischen Anomalien erfasst werden konnten: Die KIG-Kategorien U (Zahnunterzahl) und S (Durchbruchsstörungen, Retention und Verlagerung) konnten nicht beurteilt werden, da aus studienethischen Gründen keine radiologischen Aufnahmen der Kieferregion der Studienteilnehmer durchgeführt werden konnten. Da aufgrund dessen die Prävalenzen der KIG-Grade 3 bis 5, welche einen entsprechenden Versorgungsbedarf in den KIG-Kategorien U und S implizieren, nicht erhoben werden konnten, ist davon auszugehen,

dass der tatsächliche kieferorthopädische Versorgungsbedarf in der Studienpopulation der 8- und 9-Jährigen höher ist als die im Rahmen dieser Studie eruierten 40,4 %. So zeigen Studien, dass eine Prävalenz von Zahnnichtanlagen der Kategorie U von etwa 5 % angenommen werden muss und ebenso eine Prävalenz retinierter/verlagerter Zähne von etwa 6 %. Eine weitere Limitation auf methodischer Seite stellt die Verwendung der Kieferorthopädischen Indikationsgruppen (KIG) als epidemiologischer Index in einer Population von 8- und 9-Jährigen dar, während diese zur Bestimmung der Erstattungsfähigkeit kieferorthopädischer Leistungen im Rahmen der GKV-Versorgung für eine Population von über 10-Jährigen entwickelt wurde. Es besteht hier ein Risiko für eine Unterschätzung der tatsächlichen Prävalenzen und des kieferorthopädischen Versorgungsbedarfes, der 1–2 Jahre später bei der untersuchten Population im Alter von über 10 Jahren entsteht, da bekannt ist, dass die meisten kieferorthopädischen Anomalien eine Verstärkungstendenz im Wachstum zeigen [11]. Die Festlegung auf ein Kollektiv 8- und 9-Jähriger im Rahmen der DMS • 6 erfolgte jedoch bewusst, um einen möglichen Störeinfluss kieferorthopädischer Frühbehandlungen, welche oftmals vor dem 10. Lebensjahr durchgeführt werden, zu vermeiden.

Interpretation

Bezüglich der geografischen Verteilung der einzelnen Prävalenzen und KIG-Schweregrade fallen insgesamt keine relevanten Unterschiede zwischen den Subpopulationen aus Nord-, Süd-, Ost- und Westdeutschland auf. Eine Ausnahme bilden die KIG-Kategorien M (umgekehrter Frontzahnüberbiss) und K (transversale Anomalien) höherer Schweregrade, die in Süd-/Ostdeutschland tendenziell gehäuft auftreten, während sie in Norddeutschland eher unterrepräsentiert sind. Gegenteiliges zeigt sich bei der KIG-Kategorie D (vergrößerte sagittale Frontzahnstufe), welche in Norddeutschland häufiger aufzutreten scheint als in Süd-/Ostdeutschland. Auch in der Verteilung der einzelnen Prävalenzen und KIG-Schweregrade bezüglich des sozioökonomischen Status (SES) fallen insgesamt keine relevanten Unterschiede auf. Bestehende Unterschiede sind mit hoher Sicherheit auf Stichprobeneffekte aufgrund der begrenzten Fallzahl der Studie zurückzuführen.

Zukünftige Forschungsimpulse

Im Rahmen der DMS • 7 sollten die Studienteilnehmer des KFO-Moduls der DMS • 6 erneut untersucht werden, um erstmals longitudinale Daten über die Entwicklung kieferorthopädischer Anomalien mit und ohne einer in der Zwischenzeit durchgeführten kieferorthopädischen Behandlung zu erhalten. Hiermit kann u. U. auch die Wirksamkeit kieferorthopädischer Therapieverfahren evaluiert werden. Ein weiteres Augenmerk sollte in künftigen

epidemiologischen Studien auf eine reliablere Erhebung myofunktioneller Habits und Dyskinesien gelegt werden, da diese einen wesentlichen exogenen ätiologischen Faktor für die Entstehung von kieferorthopädischen Anomalien darstellen [15].

Schlussfolgerungen

In dieser Studie zur Feststellung von Zahn- und Kieferfehlstellungen wurden sowohl die deutschen Kieferorthopädischen Indikationsgruppen als auch weitere international etablierte kieferorthopädisch-epidemiologische Indizes auf das Untersuchungskollektiv 8- und 9-jähriger Kinder (frühes Wechselgebiss) angewandt, um primär bei einem möglichst großen Anteil unbehandelter Patienten den theoretischen Behandlungsbedarf festzustellen. Dabei zeigte sich, dass der kieferorthopädische Behandlungsbedarf bei 40,4 % lag, wobei zu berücksichtigen ist, dass im späten Wechselgebiss (Hauptbehandlungszeit nach den GKV-Richtlinien) aufgrund der Progredienz von Zahn- und Kieferfehlstellungen noch mit einer Zunahme zu rechnen ist und die KIG-Kategorien U (Zahnunterzahl) und S (Durchbruchsstörungen, Retention und Verlagerung) nicht berücksichtigt werden konnten. Es ist im internationalen Vergleich bei Anwendung der Richtlinien in Deutschland weder von einer Unter-, noch von einer Überversorgung auszugehen. Ein Vergleich mit den Abrechnungsdaten der Kassenzahnärztlichen Bundesvereinigung (KZBV) zeigt zudem, dass sich der kieferorthopädische Versorgungsbedarf weitgehend mit der Versorgungsrealität deckt und somit in diesem Bereich keine richtlinienspezifische Unter- oder Überversorgung anzunehmen ist.

Literaturverzeichnis

1. Alhammadi MS, Halboub E, Fayed MS et al. (2018) Global distribution of malocclusion traits: A systematic review. Dental Press J Orthod 23(6): 40.e1-40.e10. https://doi: 10.1590/2177-6709.23.6.40.e1-10.onl
2. Ruf S, Proff P, Lisson J (2021) Zahn- und Kieferfehlstellungen – gesundheitliche Relevanz und Behandlung (Health relevance of malocclusions and their treatment). Bundesgesundheitsbl 64(8): 918–923. https://doi: 10.1007/s00103-021-03372-3
3. Magalhães IB, Pereira LJ, Marques LS et al. (2010) The influence of malocclusion on masticatory performance: A systematic review. Angle Orthod 80(5): 981–987. https://doi:10.2319/011910-33.1
4. Takemoto Y, Saitoh I, Iwasaki T et al. (2011) Pharyngeal airway in children with prognathism and normal occlusion. Angle Orthod 81(1):75–80. doi:10.2319/013010-65.1
5. Maspero C, Prevedello C, Giannini L et al. (2014) Atypical swallowing: a review. Minerva Stomatol 63(6): 217–227
6. Doshi UH, Bhad-Patil WA (2011) Speech defect and orthodontics: a contemporary review. Orthodontics 12(4): 340–353.
7. Magno MB, Nadelman P, Leite KLdF et al. (2020) Associations and risk factors for dental trauma: A systematic review of systematic reviews. Community Dent Oral Epidemiol 48(6): 447–463. https://doi:10.1111/cdoe.12574
8. Batista KB, Thiruvenkatachari B, Harrison JJE et al. (2018) Orthodontic treatment for prominent upper front teeth (Class II malocclusion) in children and adolescents (Review). Cochrane Database Syst Rev 3(3): CD003452. https://doi:10.1002/14651858.CD003452.pub4
9. Lux CJ, Dücker B, Pritsch M et al. (2009) Occlusal status and prevalence of occlusal malocclusion traits among 9-year-old schoolchildren. Eur J Orthod 31(3): 294–299. https://doi:10.1093/ejo/cjn116
10. Glasl B, Ludwig B, Schopf P (2006) Prävalenz und Entwicklung KIG-relevanter Befunde bei Grundschülern aus Frankfurt am Main. J Orofac Orthop 67(6): 414–423. https://doi:10.1007/s00056-006-0615-8
11. Stahl F, Grabowski R, Gaebel M et al. (2007) Relationship between occlusal findings and orofacial myofunctional status in primary and mixed dentition. Part II: Prevalence of orofacial dysfunctions. J Orofac Orthop 68(2): 74–90. https://doi: 10.1007/s00056-007-2606-9

12. Grabowski R, Stahl F, Gaebel M et al (2007) Zusammenhang von Okklusionsbefunden und orofazialem myofunktionellem Status im Milch- und frühen Wechselgebiss: Teil I: Häufigkeit von Gebissanomalien. J Orofac Orthop 68(1): 26–37. https://doi:10.1007/s00056-007-1606-0
13. Micheelis W, Bauch J (1991) Mundgesundheitszustand und -verhalten in der Bundesrepublik Deutschland. Ergebnisse des nationalen IDZ-Survey 1989. IDZ-Materialienreihe, Bd. 11.1. Deutscher Ärzte-Verlag, Köln
14. (2003) Richtlinien des Bundesausschusses der Zahnärzte und Krankenkassen für die kieferorthopädische Behandlung in der Fassung vom 04. Juni 2003 und vom 24. September 2003 veröffentlicht im Bundesanzeiger Nr. 226 (S. 24 966) vom 03. Dezember 2003 in Kraft getreten am 1. Januar 2004
15. Turner S, Nattrass C, Sandy JR (1997) The role of soft tissues in the aetiology of malocclusion. Dental Update. 24(5): 209–214

Abbildungs- und Tabellenverzeichnis

Abb. 1 Organisation der Abläufe im Untersuchungszentrum aus Sicht der Studienteilnehmenden

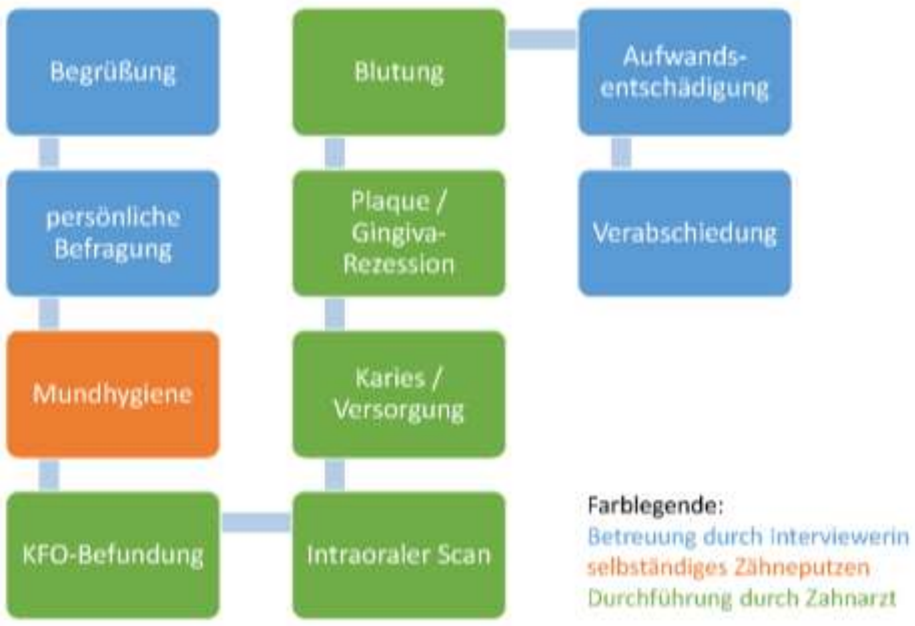

Tab. 1 Verbreitung von Habits, Dyskinesien und Dysfunktionen

		%	(95-%-KI)	n
Atemmuster	Nasenatmung	98,7	(97,5 – 99,3)	683
	Mundatmung	1,3	(0,7 – 2,5)	9
Wenn Mundatmung: Art	habituell	80,5	(48,1 – 94,9)	7
	anatomisch	19,5	(5,1 – 51,9)	2
Schluckmuster	somatisch	98,2	(97,0 – 99,0)	671
	viszeral	1,8	(1,0 – 3,0)	12
Lippenschluss	kompetent	92,2	(89,9 – 93,9)	636
	inkompetent	3,1	(2,0 – 4,7)	21
	potentiell kompetent	4,7	(3,4 – 6,6)	33
Mentalishabit		18,0	(15,4 – 21,1)	125
Zungendyskinesie: Beißen		0,4	(0,1 – 1,2)	3
Zungendyskinesie: Pressen		0,3	(0,1 – 1,1)	2
Lippendyskinesie: Saugen		2,1	(1,2 – 3,4)	14
Lippendyskinesie: Beißen		2,4	(1,5 – 3,8)	16
Lippendyskinesie: Pressen		0,2	(0,1 – 0,9)	2
Wangendyskinesie: Saugen		0,3	(0,1 – 1,1)	2
Wangendyskinesie: Beißen		13,7	(11,3 – 16,5)	95
Zwangsbiss		24,8	(21,6 – 28,2)	162
Sigmatismus oder Sprachstörung		21,9	(19,0 – 25,1)	154
Kauprobleme		6,5	(4,9 – 8,6)	46
Fingernägelkauen		26,9	(23,8 – 30,3)	190
Schlafstörungen / Schnarchen		18,1	(15,4 – 21,1)	128
Lutschdyskinesie		6,1	(4,6 – 8,1)	43

Ergebnisse der gewichteten Analyse; Rundungsabweichungen sind daher möglich.

KI = Konfidenzintervall

Tab. 2 Übersicht Kieferorthopädische Indikationsgruppen – Häufigkeitsverteilung

Indikationsgruppen	Ohne Befund	Grad 1	Grad 2	Grad 3	Grad 4	Grad 5	Gesamt
	n (%)						n
A – Kraniofaziale Anomalien	689 (99,6 %)					3 (0,4 %)	692
D – Sagittale Stufe distal		72 (10,3 %)	484 (69,2 %)		115 (16,5 %)	22 (3,2 %)	698
M – Sagittale Stufe mesial	671 (96,0 %)				24 (3,4 %)	4 (0,6 %)	698
O – Vertikale Stufe offen	653 (92,9 %)*		32 (4,6 %)	11 (1,6 %)	0 (0,0 %)	7 (1,0 %)	703
T – Vertikale Stufe tief	39 (5,7 %)	230 (33,4 %)	353 (51,2 %)	67 (9,8 %)			689
B –Bukkal-/Lingualokklusion	701 (99,7 %)				2 (0,3 %)		704
K –Kopfbiss/Kreuzbiss	644 (91,6 %)		19 (2,7 %)	3 (0,4 %)	37 (5,3 %)		704
E – Engstand	275 (39,1 %)*		364 (51,7 %)	59 (8,4 %)	5 (0,7 %)		704
P – Platzmangel	474 (69,7 %)		160 (23,5 %)	21 (3,1 %)	25 (3,6 %)		679

* Eine Unterscheidung der Kategorien „ohne Befund“ und „Grad 1“ ist mit den erhobenen Daten nicht möglich, daher werden diese Kategorien zusammengefasst angegeben.

Die Indikationsgruppen U – Zahnunterzahl und S – Durchbruchsstörungen wurden im Rahmen der Studie nicht beurteilt, da keine Röntgendiagnostik stattgefunden hat.

Ergebnisse der gewichteten Analyse; Rundungsabweichungen sind daher möglich.

Tab. 3 KIG-Schweregradeinteilung nach Geschlecht, Region und sozioökonomischem Status

KIG		Grad 1	Grad 2	Grad 3	Grad 4	Grad 5	Gesamt
		% (95-%-Konfidenzintervall)					n
Gesamt		2,5 (1,6–4,0)	57,0 (53,3 – 60,6)	10,0 (8,0 – 12,4)	25,5 (22,4 – 28,9)	5,0 (3,6 – 6,9)	705
Geschlecht	männlich	2,5 (1,3–4,6)	57,4 (52,3 – 62,4)	8,7 (6,2–12,0)	26,4 (22,1 –31,2)	5,0 (3,2–7,8)	362
	weiblich	2,6 (1,4 – 4,9)	56,5 (51,2 – 61,7)	11,3 (8,4–15,1)	24,6 (20,3 – 29,4)	4,9 (3,1–7,8)	343
Region	Norddeutschland	4,4 (2,0 – 9,5)	58,6 (49,9 – 66,8)	11,7 (7,2 –18,4)	22,6 (16,2 – 30,6)	2,7 (1,0–7,2)	127
	Süddeutschland	1,5 (0,5–4,2)	57,2 (50,4 – 63,8)	14,3 (10,2 – 19,7)	20,6 (15,6 – 26,6)	6,4 (3,8 – 10,7)	205
	Westdeutschland	1,9 (0,8–4,5)	56,9 (50,6 – 62,8)	6,0 (3,7 – 9,7)	29,9 (24,6 – 35,9)	5,3 (3,1–8,8)	249
	Ostdeutschland	3,7 (1,5–8,7)	55,3 (46,5 – 63,7)	9,0 (5,1 – 15,3)	27,8 (20,6 – 36,2)	4,3 (1,9–9,4)	124
SES	niedrig	1,5 (0,4 – 5,6)	55,4 (46,6 – 63,9)	10,1 (5,9 – 16,6)	27,20 (20,2 – 35,7)	5,7 (2,8–11,3)	124
	mittel	3,2 (1,9 – 5,6)	58,6 (53,6 – 63,6)	11,4 (8,5 – 15,0)	22,3 (18,3 –26,8)	4,5 (2,8–7,1)	370
	hoch	2,6 (0,9 – 7,2)	55,1 (46,2 – 63,6)	5,8 (2,8 – 11,4)	36,6 (28,6 – 45,4)	0,0 (0,0 – 3,0)	122

Ergebnisse der gewichteten Analyse; Rundungsabweichungen sind daher möglich. KIG = Kieferorthopädische Indikationsgruppen; SES = Sozioökonomischer Status.
